# Sex differences in COVID-19: the role of androgens in disease severity and progression

**DOI:** 10.1007/s12020-020-02536-6

**Published:** 2020-11-11

**Authors:** Mohamed S. Mohamed, Thiago C. Moulin, Helgi B. Schiöth

**Affiliations:** 1 Functional Pharmacology Unit, Department of Neuroscience, Uppsala University, Uppsala, Sweden; 2 Institute for Translational Medicine and Biotechnology, Sechenov First Moscow State Medical University, Moscow, Russia

**Keywords:** SARS-COV2, Testosterone, Nitric oxide, Hydroxychloroquine, Dexamethasone, CAG repeats

## Abstract

**Purpose:**

Throughout the SARS-CoV2 pandemic, multiple reports show higher percentages of hospitalization, morbidity, and mortality among men than women, indicating that men are more affected by COVID-19. The pathophysiology of this difference is yet not established, but recent studies suggest that sex hormones may influence the viral infectivity process. Here, we review the current evidence of androgen sensitivity as a decisive factor for COVID-19 disease severity.

**Methods:**

Relevant literature investigating the role of androgens in COVID-19 was assessed. Further, we describe several drugs suggested as beneficial for COVID-19 treatment related to androgen pathways. Lastly, we looked at androgen sensitivity as a predictor for COVID-19 progression and ongoing clinical trials on androgen suppression therapies as a line of treatment.

**Results:**

SARS-COV2 virus spike proteins utilize Transmembrane protease serine 2 (TMPRSS2) for host entry. Androgen receptors are transcription promoters for TMPRSS2 and can, therefore, facilitate SARS-COV2 entry. Variants in the androgen receptor gene correlate with androgen sensitivity and are implicated in diseases like androgenetic alopecia and prostate cancer, conditions that have been associated with worse COVID-19 outcomes and hospitalization.

**Conclusion:**

Androgen’s TMPRSS2-mediated actions might explain both the low fatalities observed in prepubertal children and the differences between sexes regarding SARS-COV2 infection. Androgen sensitivity may be a critical factor in determining COVID-19 disease severity, and sensitivity tests can, therefore, help in predicting patient outcomes.

## Introduction

The novel coronavirus disease (COVID-19) caused by severe acute respiratory syndrome coronavirus 2 (SARS-COV2) has evolved into a global pandemic and has affected millions of people worldwide. Two notably consistent findings are the low rates of prepubertal mortality [1] and that men are more likely to have severe symptoms and therefore need hospitalization [1, 2]. Moreover, sex differences in the prevalence of smoking, cardiovascular diseases, and drinking habits do not fully account for the higher risks for men [2]. Likewise, disparities between sexes have also been observed in the Middle East respiratory syndrome epidemic, where variation sex hormones were shown to rave a role in the disease susceptibility [3]. Androgens, such as testosterone and dihydrotestosterone, are steroid hormones produced in both sexes, and their levels increase with puberty. Androgens levels are higher in males than females and have been hypothesized to have a role in COVID-19 diseases [2, 4]. The interest in the role of androgens increased after the uncovering of SARS-COV2 entry points [5]. Following that, studies have shown that androgens have a role in COVID-19 disease progression and that a considerable number of hospitalized patients have an underlying androgen-mediated condition [6, 7]. In this review, we will look at how androgens facilitate SARS-COV2 entry, their role in disease progression, and their therapeutic value.

## The role of androgens is mediated by TMPRSS2

The spike proteins of SARS-COV2 intermediate the entry to host cells by undergoing spike protein priming by the transmembrane protease serine 2 (TMPSS2) and by binding to Angiotensin-converting enzyme 2 (ACE2) receptors [5]. Data from prostate cancer research has demonstrated the androgen receptor as a regulator of TMPRSS2, capable of increasing the expression of this gene [8]. For example, the TMPRSS2 plays a role in the pathophysiology of prostate cancer by interacting with the oncogenic transcription factor ERG. The interaction between these genes juxtaposes the androgen receptor elements present in their code, causing the ERG gene to be also controlled by androgen receptor signalling [9]. The androgen-dependent nature is also evident outside of the prostate, as administering exogenous androgen treatment to a human lung adenocarcinoma-derived cell line is able to increase expression of TMPRSS2, mainly in the in type II pneumocytes [10].

**Fig. 1:**
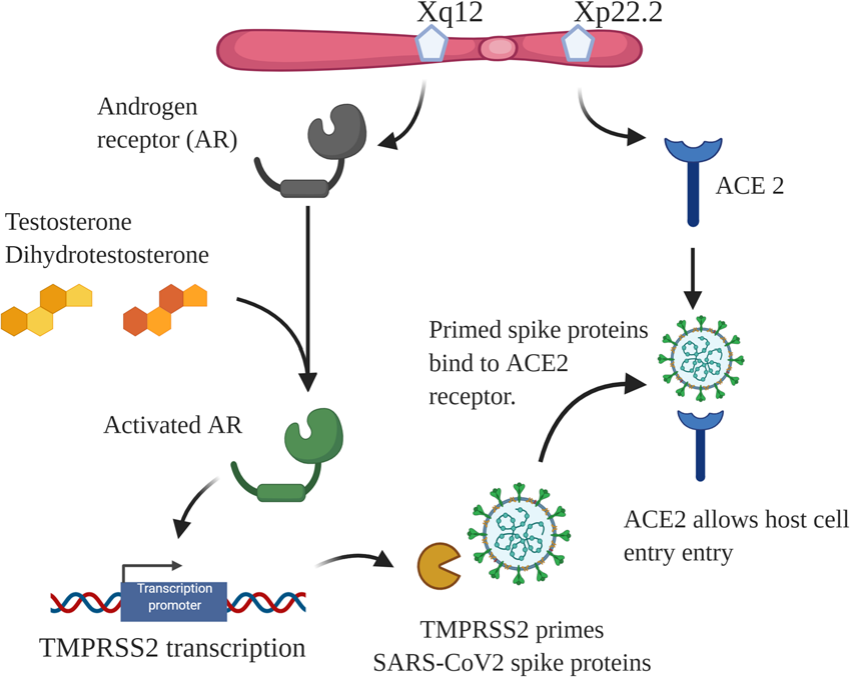
Androgens facilitate SARS-COV2 entry through a TMPRSS2-mediated pathway. Both androgen receptor and ACE2 genes are located on chromosome X. Circulating androgens binds to AR, activating it, which promotes TMPRSS2 transcription. SARS-CoV2 spike proteins are then primed by TMPRSS2, allowing the interaction with ACE2 receptors to enter host cells

Moreover, androgen deprivation therapy (ADT), a commonly-used treatment for prostate cancer patients, has been shown to lower TMPRSS2 expression [11]. The proposed mechanism behind this effect is based on the idea that androgen receptor and, subsequently, TMPPRSS2 expression affects the SARS-COV2 virus ability to enter host cells and its spike proteins affinity to bind ACE2 receptors (Fig. 1). Therefore, ADT shows the potential to provide partial protection from SARS-CoV2 infections, while measuring androgen levels might be useful for the prognosis of COVID-19 severity. Nevertheless, further pre-clinical and clinical studies are needed for a better understanding of the androgen receptor effects and possible therapeutic applications.

## Androgen sensitivity and COVID-19 disease severity

All androgens act through the androgen receptor, which is encoded by a single-copy gene on the X chromosome (Fig. 1), and variants of this gene correlate with different levels of androgen sensitivity. Such modifications increase the risk of androgen-mediated diseases such as androgenic alopecia and prostate cancer [12–14]. Evidence of androgen sensitivity association with COVID-19 disease progression can, therefore, be observed in such conditions. For example, androgenetic alopecia, a form of male pattern hair loss, is present in a substantial number of hospitalized patients [7] and was shown as a risk factor for developing severe COVID-19 symptoms [6]. Moreover, prostate cancer patients who take ADT seem to have a lower risk of COVID-19 infection compared to cancer patients without ADT [11].

However, the relationship between circulating androgen levels, androgen sensitivity, and COVID-19 severity is not straightforward. As androgens promote androgen receptor transcriptional activity, it would be expected that androgen-deprived patients would have a reduced number of activated androgen receptors to promote TMPRSS2 transcription and, thus, a decreased risk for SARS-CoV2 entry. Nevertheless, reported data from Italy and Germany suggest a contradictory outcome. Low testosterone levels can be observed in the majority of COVID-19 intensive care patients [15] and can predict poor prognosis and mortality [16]. While both studies have limitations such as lack of control groups or testosterone levels prior to infection, the results warrant consideration. Typically, androgen levels are correlated to androgen sensitivity, but many factors can affect this association [17]. For instance, although testosterone levels are known to drop with age, there is no exact threshold that predicts androgen-sensitive phenotypes, and treatments are mostly based on symptomatology [18]. A possible modulator that could also mediate SARS-CoV2 infection is inflammation. Pro-inflammatory cytokines and systemic inflammatory processes are associated with low androgens levels in young and older men [19, 20]. In addition, there is evidence that interindividual variation in androgen receptor sensitivity due to cysteine adenine guanine (CAG) polymorphisms can account for sensitivity symptoms even with ‘low’ testosterone levels [21, 22]

**Fig. 2:**
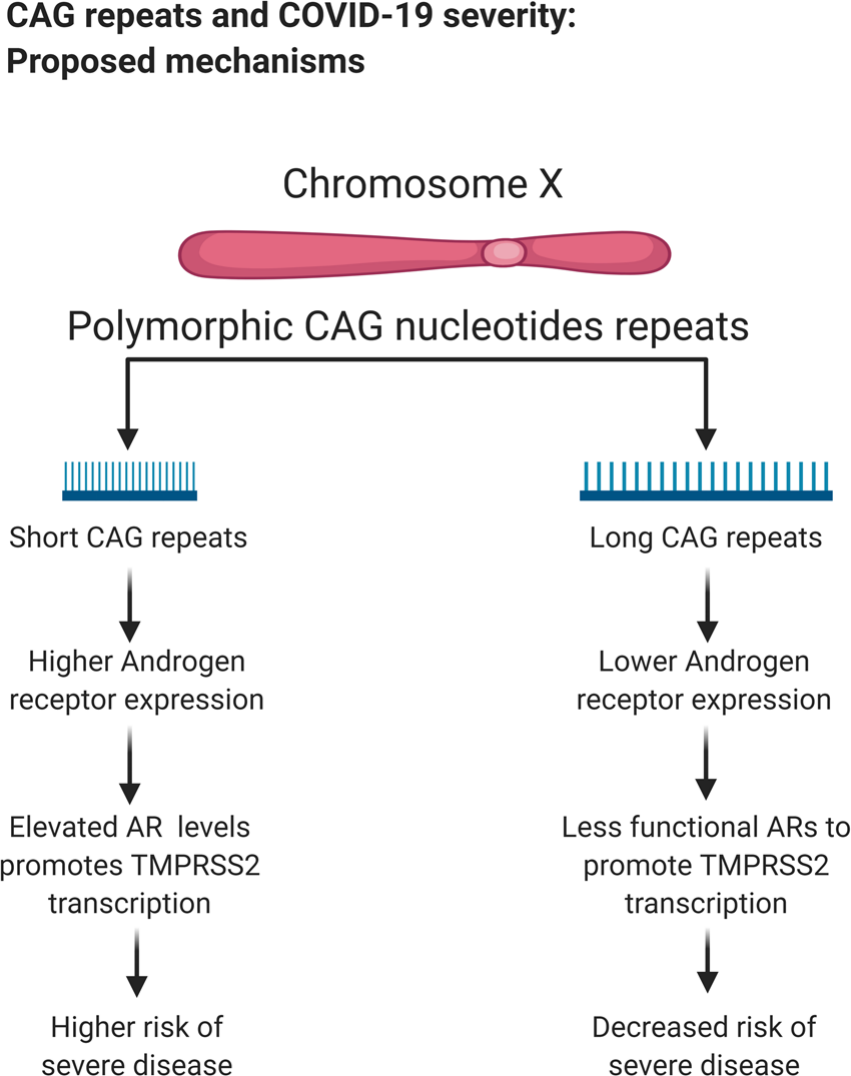
Theoretical mechanisms suggesting CAG repeats length and associated androgen sensitivity as a predictor for COVID-19 disease severity

The androgen receptor has three main functional domains: the transactivation domain, the DNA-binding domain, and the ligand-binding domain. The N-terminal transactivation domain harbors a polymorphic CAG nucleotide repeat segment. Interestingly, the length of polymorphic CAG nucleotides repeats is associated with the prostate cancer pathophysiology, as shorter CAG repeats inversely correlate to androgen receptor expression and subsequently increase the risk of prostate cancer [12]. Increased androgen receptor expression might lead to a higher risk of acquiring a severe COVID-19 disease by promoting TMPRSS2 transcription (Fig. 2). Moreover, CAG repeat length was indicated as a mechanism behind racial variations noticed for the COVID-19 mortality rate. For example, African Americans have been disproportionately affected by SARS-COV2 compared to other ethnic groups in the U.S. This ethnic group seems to have a higher risk of developing progressive prostate cancer and display shorter CAG repeats [4, 23]. In vitro diagnostic test clinical trial based on CAG repeats length is currently ongoing to evaluate COVID-19 disease severity (Table 2). It is important to notice that, to the best of our knowledge, the effects of the length of the polymorphic CAG repeat sequence in pulmonary tissue are still unknown and no clinical data are available to support this hypothesis. Thus, the results from the ongoing trials are vital for evaluating the potential of this mechanism as a COVID-19 severity marker.

## Androgen suppression targeted treatment for COVID-19

Since the start of the COVID-19 pandemic, various drugs have been proposed for treatment [24–26], but there is still no universal therapy approved. However, some medications (Table 1) have received attention due to their supposedly beneficial effects such as Hydroxychloroquine, Nitric oxide (NO), and dexamethasone. Hydroxychloroquine, an antimalarial drug, was shown to limit SARS-COV2 infections and prevent the virus entry [27, 28]. This drug was initially granted temporary FDA approval but was later revoked due to adverse effects and reported mortality [29]. However, the link to androgen’s role might be significant. There is evidence that Hydroxychloroquine can also decrease androgens secretion progressively with the duration of treatment [30].

**Table 1:** Drugs investigated as a line of treatment for COVID-19 infection, their primary targets, common indications, and proposed mechanism of action for COVID-19 treatment. Obtained from drugbank.ca

Drug	Main targets	Primary indication	Mechanism of action
Hydroxychloroquine	TLR-7, TLR-9, ACE2	Malaria prophylaxis and uncomplicated malaria	Inhibits terminal glycosylation of ACE2.
Nitric oxide	GUCY1A2, MT1A, IDO1	Hypoxic respiratory failure (neonates)	Inhibition of androgen receptors.
Dexamethasone	Glucocorticoid receptors, NR0B1, Annexin A1, NOS2, NR1I2	Bacterial infection; inflammatory conditions	Regulates testosterone synthesis.

Similarly, NO affects androgen receptor activity. NO production and actions are dependent to some extent on androgen receptors and blocking androgen receptors reduces NO production [31]. Moreover, NO decreases androgen receptor promoter actions [32], which can subsequently affect TMPRSS2 and ACE 2 expression limiting the viral ability to enter host cells. NO has been shown to suppress SARS-COV2 replication [33]. In addition, NO affects the virus spike proteins and the interactions with ACE2, suggesting a multifunctional role against COVID-19 [34]. Taken together, these results indicate that androgen pathways might be the primary mechanism behind the observed NO beneficial outcome.

Recently, preliminary results from the Randomized Evaluation of COVID-19 therapy (RECOVERY) trial have been labeled as a scientific breakthrough and received international praise due to its promising results. Dexamethasone, a glucocorticosteroid drug, reduced the mortality rate by one-third in mechanically ventilated patients and by one-fifth for those receiving oxygen without ventilation [26]. Notably, dexamethasone has been shown to lower testosterone synthesis in animal models [35, 36] and human patients [36]. Lower androgen levels might be a contributor to dexamethasone observed beneficial effects; however, these preliminary results should be interpreted with caution.

Finally, high-throughput screening to identify compounds able to reduce ACE-2 levels revealed screening hits commonly can target androgen signalling pathway. Moreover, androgen inhibitors were able to reduce ACE2 levels suggesting beneficial effects of this approach [37]. Ongoing clinical trials demonstrate the therapeutic potential of androgen suppression (Table 2). As most of these treatments are well-known and globally available, if approved, they can provide accessible and efficient COVID-19 therapies.

**Table 2:** Ongoing clinical trials registered at clinicaltrials.gov investigating the role of androgen-based therapies for COVID-19

Study title	Location	Study description	Primary outcome measures
In vitro diagnostic test to predict covid-19 mortality and disease severity	Madrid (Spain)	Androgen sensitivity test to assess the risk of developing severe SARS-CoV2 infection by AR-gene CAG repeats length	Hospital-free days to day 28 and disease severity
Hormonal intervention for the treatment in veterans with covid-19 requiring hospitalization (HITCH)	Los Angeles; New York; Washington (USA)	Phase 2 trial to assess the effects of temporary androgen suppression treatment (Degarelix) on the outcome of hospitalized veterans due to COVID-19	A composite clinical outcome at day 15 (mortality, need for hospitalization, need for mechanical ventilation)
Trial to promote recovery from covid-19 with ivermectin or endocrine therapy (RECOVER)	Baltimore (USA)	Phase 2 trial to assess recovery from COVID-19. Patients will be treated with either Ivermectin (anti-parasite), Bicalutamide (androgen blocker) or standard of care without intervention	Clinical improvement at day 7
Anti-androgen treatment for covid-19 prevention	Brasillia (Brazil)	Randomized trial to assess protective role of anti-androgen treatment (Dutasteride) for SARS-CoV2 infection	Percentage of patients hospitalized due to COVID-19 infection (within 30 days)
COVID-19 In vitro Diagnostic Test and Androgen Receptor Gene Expression	California (USA)	Cohort trial to assess the association between AR expression and COVID-19 severity using COVID-19 Androgen Sensitivity Test (CoVAST)	Percentage of patients deceased at or before 28 days
Bicalutamide to Block TMPRSS2 in Males With COVID-19 Infection	Florida, (USA)	Phase 3 trial to assess the effects of Bicalutamide (anti-androgen) to block TMPRSS2 and clinical outcome of patients	Percentage of improved patients at day 28
Enzalutamide Treatment in COVID-19 (COVIDENZA\)	Malmo, Jönköping (Sweden)	Phase 2 trial to assess the effects of short-term Enzalutamide (anti-androgen) treatment of COVID-19 patients	Clinical outcome assessed by the 7-point ordinal scale (Up to 30 days)

In summary, androgen’s TMPRSS2-mediated actions can explain both the low fatalities observed in prepubertal children and the differences between sexes regarding SARS-COV2 infection. Androgen sensitivity might be a critical factor in determining COVID-19 disease severity, and sensitivity tests can, therefore, help in predicting patient outcomes. There is still a large potential for development of androgen suppression-based treatments for COVID-19, but ongoing trials will provide valuable knowledge that can lead to improved therapies.
